# Speech and sMRI as multimodal biomarkers for early detection of Alzheimer’s disease using machine learning, deep learning, and transformer-based models

**DOI:** 10.3389/fnagi.2026.1975870

**Published:** 2026-10-27

**Authors:** Vyshnavi Ramineni, Faizaan Fazal Khan, Jun-Hyung Kim, Goo-Rak Kwon

**Affiliations:** Department of Information and Communication Engineering, Chosun University, Gwangju, Republic of Korea

**Keywords:** Alzheimer’s disease, mild cognitive impairment, multimodal fusion, speech biomarkers, structural MRI, transformer models

## Abstract

Alzheimer’s disease (AD) and its prodromal stage, MCI are typically diagnosed using cerebrospinal fluid analysis and amyloid positron emission tomography procedures that are invasive, costly, and poorly suited to population-scale screening. Speech and structural magnetic resonance imaging (sMRI) have emerged as complementary non-invasive alternatives: speech captures the real-time functional consequences of neurodegeneration on lexical retrieval, semantic organization, and prosody, while structural MRI reveals the anatomical substrate of cortical and subcortical atrophy that underlies these impairments. This Mini Review examines how machine learning, deep learning, graph based and transformer based models have been applied to these two modalities, individually and in combination, for classifying cognitively normal individuals and those with MCI or AD. We trace the progression from classical machine learning on hand-engineered acoustic and lexical features, through convolutional and recurrent neural networks that learn volumetric atrophy patterns and discourse-level speech representations, to vision transformers and graph neural networks that capture long-range spatial and connectomic dependencies in brain structure. We then examine multimodal fusion strategies early, late, intermediate and the cross-modal attention architectures that link speech and MRI within a single, interpretable framework. While intermediate fusion shows the strongest diagnostic performance in early evidence, this advantage narrows in the three-way classification task most relevant to clinical screening. We close by identifying dataset diversity, language dependency, and the lack of longitudinal multimodal studies as the principal barriers to clinical translation.

## 1 Introduction

Alzheimer’s disease (AD) is the leading cause of dementia globally. In 2021, AD and other dementias caused over 8 million deaths and more than 25 million disability-adjusted life years among adults aged 65 years and older across 204 countries (Xiaopeng et al., 2025). In the United States alone, an estimated 6.9 million individuals currently live with AD dementia, a figure projected to double to 13.8 million by 2060 (2024 Alzheimer’s disease facts and figures, 2024). Globally, total case numbers increased by 141.25% between 1992 and 2021 (Qin et al., 2025).

The pathophysiological cascade of AD precedes clinical symptom onset by decades. Longitudinal Alzheimer’s Disease Neuroimaging Initiative (ADNI) data (Weiner et al., 2017) show that amyloid alterations are detectable approximately 5 years before the amyloid-positivity threshold, followed by neurodegeneration and cerebrospinal fluid tau elevation, with measurable cognitive decline emerging within 2 years thereafter (Schaap et al., 2024). Among individuals with mild cognitive impairment (MCI), 42.2% convert to AD dementia, and integrating amyloid PET with structural MRI achieves an AUC of 0.891 for predicting this conversion (Kim, 2025).

Current gold-standard diagnostic tools present substantial barriers to population-scale early detection. Cerebrospinal fluid collection (Mazzeo et al., 2024) requires invasive lumbar puncture, and amyloid PET demands cyclotron infrastructure inaccessible in lower-resource settings, (Leuzy et al., 2025). Hippocampal atrophy on structural MRI demonstrates 89% sensitivity for predicting clinical progression and, when combined with plasma biomarkers, discriminates fast from slow progressors in MCI with area under the curve (AUC) values of 0.78–0.93 (Xie et al., 2023; Manjavong et al., 2024).

Two non-invasive signals have emerged as compelling complements to neuroimaging for scalable early detection: acoustic features of spontaneous speech, including filled pause frequency reflecting impaired lexical retrieval, and speech-derived linguistic markers such as reduced function word use and altered content density, which correlate with structural brain changes on MRI in MCI due to AD (Lee et al., 2025). This Mini Review therefore examines the evidence applied to speech and sMRI for the classification of cognitively normal individuals, those with MCI, and those with AD, with a focus on the neurobiological basis of each modality, the computational approaches employed, and the potential gains achievable through multimodal integration.

## 2 Speech as a marker of cognitive decline in Alzheimer’s disease

Speech based biomarkers of AD encompass acoustic, linguistic, and discourse-level features associated with cognitive decline. Advances in machine learning, deep learning, and transformer-based models, alongside novel representations of discourse organization, have expanded their potential for early detection and integration with sMRI.

### 2.1 Acoustic and linguistic manifestations of neurodegeneration

Fluent, coherent speech requires the coordinated engagement of lexical retrieval from the left temporal lobe, semantic processing dependent on hippocampal-entorhinal circuitry, syntactic planning governed by the left inferior frontal gyrus, and articulatory execution via motor cortical pathways. Because these regions are selectively and sequentially vulnerable to the neuropathological changes of AD, the spoken language of individuals with MCI and AD degrades in characteristic and measurable ways that mirror the underlying pattern of neurodegeneration (Braak and Braak, 1991; Jack et al., 2024).

The earliest and most consistently reported speech changes in MCI reflect disruption to hippocampal-dependent lexical access: prolonged and more frequent silent pauses indicate a slowing of word retrieval from impaired temporal-hippocampal memory networks (Chandler et al., 2023; Ding et al., 2024). As pathology progresses toward clinical AD, reductions in speech rate and perturbations in prosodic rhythm reflect the extension of neurodegeneration into motor speech planning networks, progressing in a temporospatial gradient that mirrors the Braak staging framework.

At the linguistic level, individuals with MCI characteristically produce speech with reduced information unit density, generating more words to convey less meaningful content reflecting progressive disorganization of semantic knowledge networks in the anterior temporal lobe (Lima et al., 2025). Syntactic complexity declines as embedded-clause construction exceeds the capacity of the prefrontal-parietal executive network, and pronoun production increases while noun generation decreases, reflecting vulnerability of confrontation naming to hippocampal and lateral temporal atrophy (Kovac et al., 2025). These linguistic markers correlate with structural brain changes on MRI in MCI due to AD, with reduced function word use and altered content density mapping onto cortical thinning in frontal and temporal regions.

The principal elicitation paradigm for speech biomarker research is the Cookie Theft picture description task. The Dementia Bank Pitt Corpus (MacWhinney, 2019), the ADReSS Challenge dataset (Luz et al., 2020), and the Framingham Heart Study speech corpus are the primary community benchmarks against which computational methods have been validated.

### 2.2 Machine learning and deep learning approaches for speech-based classification

Classical machine learning approaches established that automated analysis of connected speech captures diagnostically meaningful information about the underlying neural substrate vector machines and random forest classifiers, trained on acoustic pause and lexical semantic features, consistently discriminated AD from controls, with the most informative features aligning with temporal lobe-dependent lexical retrieval and frontal executive discourse planning (Chandler et al., 2023; Lima et al., 2025). Filled pause frequency was the most discriminative acoustic feature for MCI detection in the Framingham Heart Study, reflecting hippocampal atrophy and reduced connectivity between temporal memory systems and frontal language networks (Ding et al., 2024); its detectability from non-specialized voice recordings underscores its potential as a scalable screening indicator.

Recurrent neural network/ Long short-term memory (RNN/LSTM) architectures and spectrogram-based convolutional neural networks (CNNs) capture, respectively, disrupted prosodic rhythm and reduced articulatory precision in later disease stages Table 1. When deep learning models trained on Framingham Heart Study speech transcripts were evaluated for their ability to predict long-term neurodegenerative trajectory, the linguistic patterns (Amini et al., 2024) followed 166 Framingham Heart Study participants had MCI and asked whether their speech could predict progression to AD within 6 years. Using language-model embeddings of transcribed speech with logistic regression, they found that speech carried prognostic information beyond demographic factors. This suggests that connected speech may hold information about future decline that standard neuropsychological scores do not fully capture. Multimodal deep learning combining acoustic and linguistic streams outperforms either modality alone, since each captures partially independent dimensions of AD-related decline (Lin and Washington, 2024).

**Table 1:** Computational approaches applied to speech and structural MRI for AD/MCI classification, organized by modality, approach, and architecture.

Modality	Approach	Architecture(s)	Key finding	Reference
Speech	Machine learning	SVM, random forest	Discriminated AD from controls using acoustic pause and lexical-semantic features	Chandler et al. (2023) and Lima et al. (2025)
Speech	Machine learning	Feature-based classifiers	Filled pause frequency was the most discriminative feature for MCI detection (AUC 0.87, Framingham cohort)	Ding et al. (2024)
Speech	Deep learning	Transcript embeddings + logistic regression	Used speech-derived language representations to investigate six-year progression among 166 participants with MCI	Amini et al. (2024)
Speech	Multimodal DL	Acoustic + linguistic fusion	Combining acoustic and linguistic features improved classification compared with individual speech modalities.	Lin and Washington (2024)
Speech	Graph-based discourse analysis	Word-level speech graphs	Participants with AD produced less connected narratives, and reduced connectedness was associated with poorer semantic memory	Malcorra et al. (2021)
Speech	Graph-based connectedness analysis	Speech graph representation	Greater speech connectedness was associated with better cognitive functioning in English-speaking participants.	Botezatu et al. (2023)
Speech	Deep learning and conversational analysis	Topological-kinetic model (2TK) + CNN	Topological-kinetic representations of conversational dynamics were used for CNN-based AD classification.	Trognon et al. (2025)
Speech	Transformer	BERT (text)	Transformer-based analysis identified discourse abnormalities not captured by conventional lexical-frequency features	Ilias et al. (2023)
Speech	Transformer	wav2vec + BERT (cross-modal)	Combined acoustic and linguistic representations captured complementary speech characteristics relevant to AD classification.	Zhu et al. (2021)
MRI	Deep learning	3D CNN	Captured continuous hippocampal/entorhinal atrophy patterns missed by 2D slice methods	ur Rahman et al. (2025) and Zilioli et al. (2026)
MRI	Deep learning	CNN + Grad-CAM	Attribution maps identified hippocampus, entorhinal cortex, amygdala as most discriminative regions	El-Assy et al. (2024) and Veitch et al. (2024)
MRI	Deep learning (ensemble)	DenseNet201, ResNet50, Inception-ResNet	Parallel backbones better captured heterogeneous atrophy subtypes	Komal et al. (2025) and Zilioli et al. (2026)
MRI	Deep learning	RNN/LSTM (longitudinal)	Modeled atrophy trajectory across sequential scans rather than single timepoints	El-Assy et al. (2024)
MRI	Transformer	Vision Transformer (Swin Transformer)	Hierarchical attention captured long-range dependencies; attention maps aligned with known Braak-stage vulnerability	Alp et al. (2024) and Zhao et al. (2024)
MRI	Graph-based	Graph neural network	Modeled brain as connectome; identified hub regions whose degeneration disrupts the most network connections	Qu et al. (2023) and Shen et al. (2025)
Multi-modal	Transformer (cross-modal)	Cross-modal attention (speech + MRI)	Higher attention weight on pause duration and lexical diversity in participants with medial temporal lobe atrophy	Duong et al. (2025)
Multi-modal	Transformer (surface)	Structural + FDG-PET cross-attention	Aligned structural atrophy with metabolic decline at the cortical surface level	Latif et al. (2025) and Han (2025)

### 2.3 Graph based and conversational analysis of connected speech

Beyond conventional acoustic and linguistic features, discourse organization and connectedness have emerged as complementary speech biomarkers for AD. Malcorra et al. (2021) reported reduced narrative connectedness associated with poorer semantic memory in Brazilian Portuguese-speaking individuals with AD, while (Botezatu et al., 2023) identified associations between speech connectedness and cognitive functioning in English-speaking participants. Trognon et al. (2025) further investigated French language conversations using topological kinetic representations and CNN-based classification. Together, these studies highlight the importance of behavioral representations beyond conventional speech features and classifier architectures, while demonstrating the potential of discourse based approaches across different languages.

### 2.4 Transformer based models and their clinical relevance

Transformer-based models provide a complementary computational approach to analyzing discourse organization by capturing long range linguistic dependencies and contextual relationships across extended speech. Self-attention mechanisms are well suited to the clinical phenomenology of AD-related language impairment, since the disease disrupts referential coherence across extended discourse precisely the long-range dependencies transformers are designed to capture (Ilias et al., 2023). Bidirectional Encoder Representations from Transformers (BERT)-applied to speech transcripts detect AD-related discourse abnormalities missed by lexical-frequency approaches Table 1. The self-supervised wav2vec architecture, pre-trained on unlabeled speech, learns representations sensitive to the pause patterns and voice quality changes that reflect hippocampal and basal ganglia involvement in AD, without manual feature engineering (Zhu et al., 2021). When wav2vec’s acoustic representations are aligned with BERT’s linguistic representations through a cross-modal architecture, the resulting system jointly captures the semantic and prosodic impoverishment of AD speech.

Transformer attention weights can be visualized to show which words or discourse segments drove a classification an interpretability advantage discussed further below (Ilias et al., 2023; Chlasta et al., 2025). A clinician cannot act on a black-box prediction but can act on an explanation highlighting the specific patterns of lexical emptiness and discourse fragmentation that correspond to deficits the clinician already understands, making transformer-based speech analysis a genuinely clinically deployable window on the neurodegenerative process. Neuroimaging biomarkers and deep learning models in AD.

### 2.5 Structural and functional neuroimaging correlates of AD pathology

The neuropathological process of AD begins in the medial temporal lobe, years to decades before any cognitive symptom is detectable. The sequential deposition of tau neurofibrillary tangles along the transentorhinal pathway, described in the Braak staging framework, produces a spatially predictable gradient of neurodegeneration from the entorhinal cortex through the hippocampus into the neocortex gradient directly legible on structural MRI (Braak and Braak, 1991).

Hippocampal volume reduction is the most extensively validated structural correlate of AD-related neurodegeneration, with longitudinal ADNI data confirming that atrophy trajectories diverge between individuals who remain cognitively stable and those who progress from MCI to AD dementia (Xie et al., 2023; Schaap et al., 2024). Hippocampal volume alone does not capture the full anatomical scope of early neurodegeneration entorhinal cortex thinning and para hippocampal subfield atrophy reflect advancing tau pathology in Braak stages III through VI, and their combination provides substantially greater discriminative information than any single region (Xie et al., 2023).

Resting-state fMRI reveals a complementary dimension of AD-related pathology that structural imaging cannot capture. The default mode network shows early disruption of effective connectivity in individuals who will later develop dementia, with dynamic causal modelling of resting-state fMRI in the UK Biobank showing that DMN dysconnectivity predicted future dementia incidence up to 9 years before clinical diagnosis (Ereira et al., 2024). This sequential pattern functional changes preceding structural atrophy, which itself precedes cognitive symptoms is the biological rationale for combining resting-state (functional MRI) fMRI with sMRI and speech-based markers in a multimodal framework (Ereira et al., 2024; Schaap et al., 2024).

### 2.6 Convolutional and recurrent neural networks for neuroimaging classification

Three-dimensional CNN architectures capture the spatially continuous patterns of hippocampal and entorhinal atrophy that characterize medial temporal lobe involvement in early AD, which 2D slice-based approaches fragment by treating each axial section as independent (ur Rahman et al., 2025; Zilioli et al., 2026). Grad-CAM attribution applied to trained CNN classifiers consistently identifies the hippocampus, entorhinal cortex, amygdala, and lateral ventricles as the anatomical loci driving classification decisions precisely the regions of earliest and most severe neurodegeneration in the Braak staging framework (El-Assy et al., 2024; Veitch et al., 2024) Ensemble architectures combining multiple CNN backbones Table 1 better capture the heterogeneity of atrophy patterns across AD subtypes than any single architecture (Komal et al., 2025; Zilioli et al., 2026).

Transfer learning extends deep CNN approaches to smaller cohorts, though models pre-trained on other neuroimaging cohorts transfer atrophy-sensitive features more reliably than those pre-trained on non-medical images (Veitch et al., 2024). This has motivated recurrent architectures particularly LSTM networks that model disease progression across sequential MRI acquisitions, capturing the rate and trajectory of atrophy change most informative for distinguishing stable MCI from individuals progressing toward AD dementia (El-Assy et al., 2024).

### 2.7 Vision transformers and graph neural networks for brain image analysis

Because AD pathology spreads across anatomically distant but connectionally coupled regions, architectures that model long-range spatial dependencies are well suited to capturing its full anatomical scope (Zhao et al., 2024; Zhou et al., 2025). Vision transformers, which divide the volumetric MRI scan into non-overlapping three-dimensional patches and apply self-attention mechanisms to model relationships between all pairs of patches simultaneously, naturally capture these long-range dependencies. In the Swin Transformer architecture, a hierarchical shifted-window attention mechanism progressively integrates information across spatial scales from local patch-level features that correspond to hippocampal subfield atrophy to global brain-level patterns reflecting distributed network degeneration (Zhou et al., 2025). When applied to T1-weighted ADNI MRI data, Vision Transformer architectures have demonstrated that the attention maps generated during inference are interpretable through the lens of known AD neuropathology the regions receiving highest attention weight correspond systematically to the medial temporal lobe structures most vulnerable in early Braak stages, providing clinicians with spatially explicit evidence for the model’s diagnostic reasoning rather than an opaque classification label (Alp et al., 2024; Zhao et al., 2024).

Graph neural networks (GNN) model the brain as a network cortical and subcortical regions as nodes, white matter tracts or functional connectivity as edges rather than a Euclidean volume. A graph convolutional network trained on AD-relevant biomarkers weights each region’s contribution by both local atrophy and its position within the connectomic architecture, identifying hub regions whose degeneration disrupts the most network connections, the pattern of hub vulnerability that characterizes the most clinically severe AD cases (Qu et al., 2023; Shen et al., 2025).

## 3 Multimodal fusion of speech and neuroimaging for CN, MCI, and AD classification

### 3.1 Fusion strategies and their biological rationale

Speech and sMRI provide complementary information about AD pathology: MRI reveals anatomical changes, while speech reflects the functional consequences of neurodegeneration (Ilias et al., 2023; Liu et al., 2024). A critical biological observation motivating multimodal fusion is the temporal relationship between structural and functional markers of early disease. The default mode network (DMN) comprises interconnected brain regions, including the posterior cingulate cortex, precuneus, medial prefrontal cortex, and lateral parietal cortex, that support memory and internally directed cognition (Menon, 2023). Altered DMN functional connectivity is associated with AD, although the direction and extent of these changes may vary across disease stages (Seoane et al., 2024). Resting-state fMRI studies have identified changes in DMN effective connectivity years before clinical dementia diagnosis (Ereira et al., 2024), while speech alterations may provide complementary indicators of cognitive decline. Together, these findings provide a biological rationale for investigating the integration of speech and neuroimaging biomarkers for early AD detection.

Three principal fusion strategies have been explored: early fusion (concatenating features before modeling), late fusion (combining independently trained classifiers’ outputs), and intermediate fusion (combining modality-specific representations via cross-modal attention or shared latent space learning) Figure 1. Intermediate fusion has consistently shown superior performance, suggesting the most diagnostically useful information emerges from cross-modal interaction rather than either modality alone (Guo et al., 2025).

**Figure 1:**
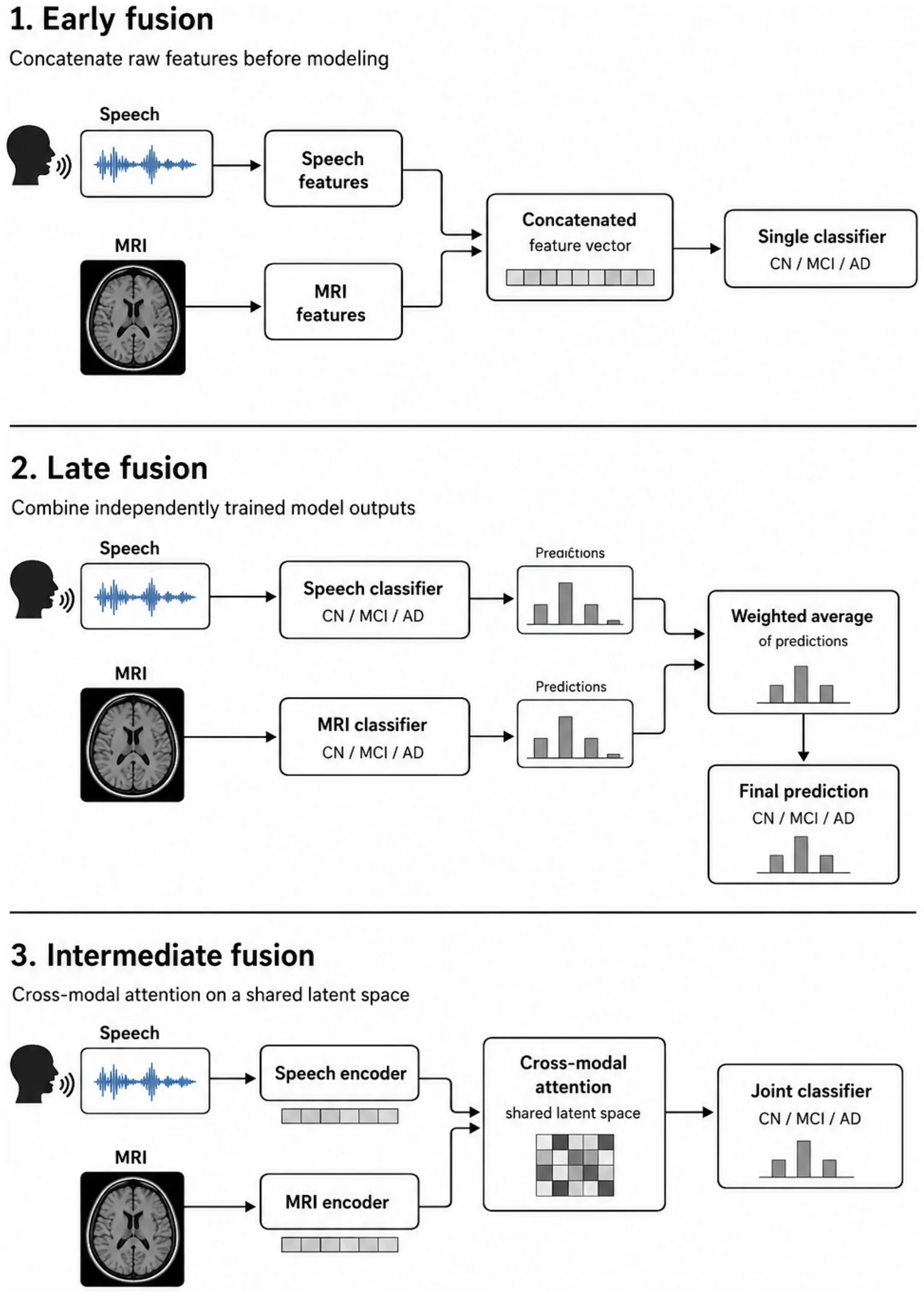
Three multimodal fusion strategies for combining speech and structural MRI: 1. Early fusion (feature concatenation), 2. Late fusion (combining classifier outputs), 3. Intermediate fusion (cross-modal attention on a shared latent space).

### 3.2 Cross-modal transformer architectures and interpretability

Cross-modal attention mechanisms, which allow the computational model to dynamically weight the contribution of features from one modality based on the contextual information provided by another, represent the natural realization of the biological principle that speech and brain structure are not independent biomarkers but co-varying expressions of a shared underlying neuropathological process. Cross-modal attention mechanisms dynamically weight speech features according to a participant’s MRI-derived atrophy pattern. The result higher attention weight on pauses duration and lexical diversity in participants with pronounced medial temporal lobe atrophy reflects the known circuit-level link between hippocampal neuronal loss and the impairment of lexical retrieval that produces the most diagnostically salient speech changes in early AD (Duong et al., 2025).

Surface-based transformer models applied to cortical MRI data use cross-attention between structural surface features derived from T1-weighted scans and metabolic features derived from fluorodeoxyglucose PET (FDG-PET) to identify the spatial distribution of functional metabolic decline relative to structural atrophy a relationship that mirrors the Braak staging model at the cortical surface level, with tau-vulnerable regions showing the earliest co-occurrence of structural loss and metabolic disruption (Duong et al., 2025). Extended to incorporate speech as a third modality, such architectures link brain structure, brain function, and language output within a single framework (Latif et al., 2025; Han, 2025).

This interpretability has clinical significance: cross-modal attention maps reveal which speech segments and brain regions the model weighted most heavily, and crucially, whether these coincide with the patterns of atrophy and discourse impairment the attending clinician would independently identify as significant (Latif et al., 2025; Xie et al., 2026). This is not an aesthetic property of these models; it is a clinical necessity. A model that reproduces the clinician’s own suspected findings, quantitatively and from non-invasive data, provides genuinely actionable support; one that cannot be interrogated provides, at best, an uncontextualized probability (Guo et al., 2025).

## 4 Discussion

This review’s evidence indicates that both speech and sMRI are biologically grounded markers of AD-related neurodegeneration, with computational approaches progressing from classical machine learning to three-dimensional convolutional networks, vision transformers, and cross-modal attention architectures (Afifi et al., 2025; Guo et al., 2025; Lima et al., 2025).

However, important tensions remain. The question of whether speech or MRI is the more reliable early biomarker is unsettled. Resting-state fMRI predicts dementia incidence up to 9 years before clinical diagnosis (Ding et al., 2024), a temporal advantage speech-based approaches have not yet demonstrated in comparably large prospective cohorts; conversely, speech data is dramatically easier and cheaper to collect, requiring no specialized scanning equipment or radiation exposure, giving it a scalability advantage MRI cannot match at the population level. Whether multimodal fusion consistently outperforms the better unimodal approach is also unresolved: intermediate fusion outperforms early or late fusion in meta-analytic evidence, but this is based predominantly on binary CN-versus-AD classification, and the advantage narrows substantially in the clinically critical three-way CN/MCI/AD task that is the true target of early detection (Guo et al., 2025).

Several research gaps must be addressed before clinical translation. The ADNI cohort, which underpins most MRI-based deep learning studies, and the Dementia Bank Pitt Corpus and Framingham Heart Study, which anchor most speech biomarker research, are overwhelmingly composed of non-Hispanic white, highly educated participants from North American academic centers (Battineni et al., 2024; Veitch et al., 2024). models trained on these cohorts may not generalize to the populations bearing the greatest AD burden. Although English-language datasets dominate speech-based AD research, studies involving non-English-speaking populations have also emerged. Malcorra et al. (2021) investigated graph-based discourse connectedness in Brazilian Portuguese, while Trognon et al. (2025) examined conversational organization in French. Nevertheless, acoustic and linguistic markers may differ across languages, highlighting the need for further multilingual validation (Kim et al., 2024). Class imbalance, MCI is typically underrepresented relative to CN and AD and the near-absence of longitudinal multimodal studies tracking the same individuals over years mean the temporal relationship between speech deterioration and structural atrophy remains largely hypothesized rather than demonstrated (Xie et al., 2023; Lima et al., 2025).

Future research should prioritize several directions. Federated learning frameworks, which distribute model training across institutions without sharing raw data, offer a path toward larger and more diverse multimodal datasets while preserving participant privacy (Guo et al., 2025). Multilingual, self-supervised speech models and discourse-based representations offer promising directions for extending AD detection beyond English, building on existing non-English studies (Malcorra et al., 2021; Chlasta et al., 2025; Trognon et al., 2025). The integration of resting-state fMRI with speech analysis represents an underexplored direction with strong biological motivation, if DMN effective connectivity disruption predicts dementia 9 years before diagnosis (Ereira et al., 2024) and speech changes reflect the functional consequences of that same network disruption, combining these signals in a principled multimodal framework could extend the detection window substantially beyond what structural MRI or speech analysis alone can achieve. Finally, and most critically, the field requires prospective clinical validation trials in which computational models trained on research cohorts are prospectively evaluated in real clinical workflows, with outcomes assessed against gold-standard biomarker confirmation and adjudicated clinical diagnosis without such validation, the high classification performances reported in retrospective benchmark studies remain promises rather than evidence of clinical utility.
